# Late gadolinium enhancement cardiovascular magnetic resonance for sudden cardiac death risk stratification in hypertrophic cardiomyopathy

**DOI:** 10.1186/1532-429X-15-S1-O67

**Published:** 2013-01-30

**Authors:** Tevfik F Ismail, Andrew Jabbour, Ankur Gulati, Amy Mallorie, Sadaf Raza, Thomas E Cowling, Bibek Das, Jahanzaib Khwaja, Rick Wage, James Moon, Amanda Varnava, Carl Shakespeare, Perry Elliott, Rory OHanlon, Dudley J Pennell, Sanjay K Prasad

**Affiliations:** 1 CMR Unit & NHLI Imperial College London, Royal Brompton Hospital & NHLI Imperial College London, London, UK; 2 Institute of Cardiovascular Science, Heart Hospital & University College London, London, UK; 3 Cardiology, West Hertfordshire Hospitals NHS Trust, Watford, UK; 4 Cardiology, South London Hospitals NHS Trust, London, UK

## Background

Although myocardial fibrosis identified by late gadolinium enhancement (LGE) cardiovascular magnetic resonance (CMR) may predict adverse outcomes among patients with hypertrophic cardiomyopathy (HCM), its precise role in risk stratification for sudden cardiac death (SCD) remains unresolved. Previous studies have relied on broad surrogate composite endpoints and were underpowered to assess SCD risk or to adjust for confounding variables. To address this, we studied the prognostic significance of LGE in a large HCM cohort with long-term follow-up.

## Methods

Consecutive patients with HCM (n=711, median age 56.3 years, interquartile range [IQR] 46.7 to 66.6; 70.0% male) underwent LGE-CMR and were prospectively followed for a median of 3.5 years. This amounted to a total of 2852 patient-years of follow-up. The primary endpoint was SCD or aborted SCD. The principal secondary endpoint was a composite of cardiovascular mortality and aborted SCD. LGE imaging was performed in two orthogonal phase-encoding directions ~10 min after 0.1 mmol/kg gadolinium using an inversion recovery-prepared gradient echo sequence. The amount was quantified using the full-width at half-maximum method.

## Results

**Figure 1:**
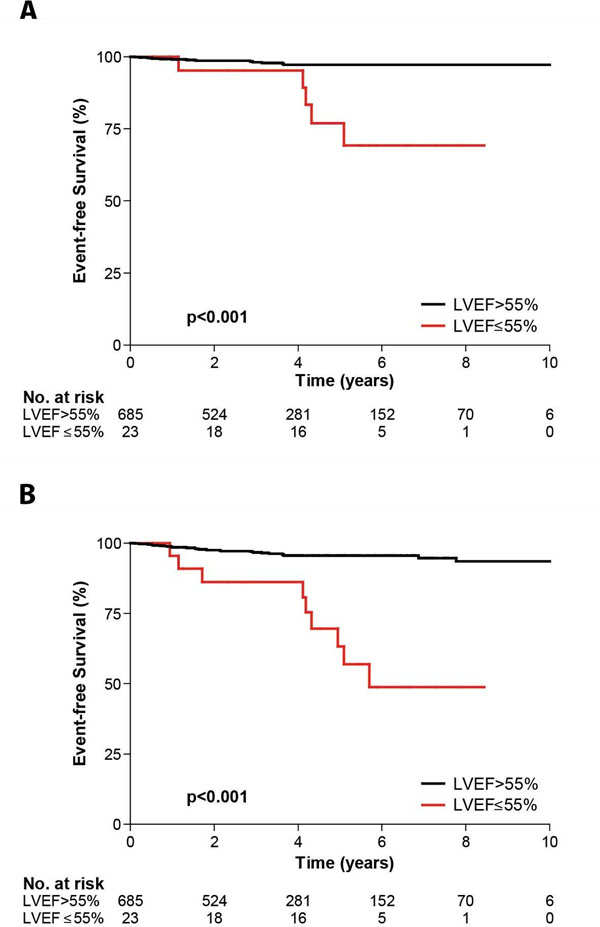
Kaplan-Meier survival estimates for A) sudden cardiac death or aborted sudden cardiac death; and B) cardiovascular mortality of aborted sudden cardiac death stratified by left ventricular ejection fraction.

Patients with LGE had more significant left ventricular hypertrophy and lower left ventricular ejection fraction (LV-EF) than those without LGE (Table 1). The median amount of LGE in the 471 (66.2%) patients with enhancement was 5.9% of left ventricular mass (IQR: 2.2 to 13.3). At the end of follow-up, 22 patients (3.1%) reached the primary endpoint: 18 (3.8%) in the LGE group and 4 (1.7%) in the no LGE group. The amount but not the presence of fibrosis was a significant univariate predictor of SCD risk (HR per 5% LGE: 1.24, 95% CI 1.06 to 1.45; p=0.007 and HR for LGE: 2.69, 95% CI 0.91 to 7.97; p=0.073 respectively). However, on multivariate analysis, only left ventricular ejection fraction (LV-EF) remained significant (HR: 0.92, 95% CI 0.89 to 0.95; p<0.001)[Figure 1]. For the secondary composite endpoint of cardiovascular mortality and aborted SCD, 30 (6.4%) patients in the LGE group versus 8 (3.3%) in the no LGE group reached the endpoint (HR for LGE: 2.24, 95% CI 1.03 to 4.89; p=0.043). However, on multivariate analysis, only LV-EF and non-sustained VT (NSVT) emerged as a significant predictors of outcome (HR for LV-EF: 0.92, 95% CI 0.90 to 0.95, P<0.001; and HR for NSVT: 3.15, 1.37 to 7.28, p=0.007).

**Table 1:** 

CMR parameters	No LGE (n=240 [33.8%])	LGE (n=471 [66.2%)	All patients (n=711)	P value
**LV-EDV index - ml/m^2^**	67.8±14.5	69.8±16.2	69.1±15.7	0.123
**LV-ESV index - ml/m^2^**	15.9±7.5	19.3±10.3	18.2±9.6	<0.001
**LV ejection fraction - %**	77.1±7.3	73.3±9.5	74.6±9.0	<0.001
<50%	1 (0.4%)	11 (2.3%)	12 (1.7%)	0.021
50-59%	5 (2.1%)	25 (5.3%)	30 (4.2%)	
≥60%	232 (97.5)	434 (92.3)	666 (94.1%)	
**LV mass index - g/m^2^**	88.7±25.1	108.4±40.1	101.9±37.0	<0.001
**Maximum end-diastolic wall thickness - mm**	16.6±3.7	20.8±5.2	19.4±5.1	<0.001

LV=Left Ventricular; LV-EDV=Left ventricular End-Diastolic Volume; LV-ESV=Left Ventricular End-Systolic Volume.

## Conclusions

Neither the presence nor the amount of LGE independently predicted SCD risk after adjusting for confounders. In contrast, LV-EF was the best independent predictor of SCD and cardiovascular mortality, and should therefore be considered as part of the routine risk stratification of patients with HCM.

## Funding

This work is supported by the NIHR Cardiovascular Biomedical Research Unit at the Royal Brompton and Harefield NHS Foundation Trust, and Imperial College. Dr Ismail is supported by the British Heart Foundation.

